# CD5+ Diffuse Large B-Cell Lymphoma With Leukemic Transformation: A Rare Case With Central Nervous System Involvement, Treated With R-CHOP and Intrathecal Methotrexate/Cytarabine

**DOI:** 10.7759/cureus.15838

**Published:** 2021-06-22

**Authors:** Justin Cordova, Blessie Nelson, Ashley M Brizendine, Fatima Iqbal, Rohit Venkatesan

**Affiliations:** 1 Department of Hematology and Oncology, University of Texas Medical Branch, Galveston, USA; 2 Department of Pathology and Laboratory Medicine, University of Texas Medical Branch, Galveston, USA; 3 Department of Oncology, University of Texas MD Anderson Cancer Center, Galveston, USA

**Keywords:** leukemic transformation, non-hodgkin's lymphoma, r-chop therapy, dlbcl, cd5+

## Abstract

Diffuse large B-cell lymphoma (DLBCL) is one of several subtypes of non-Hodgkin’s lymphoma, and one that can present in a myriad of ways. One unique and particularly aggressive presentation is leukemic transformation with CD5 positivity, which leads to systemic symptoms, a relatively high peripheral tumor load, and higher rates of CNS involvement. The prevalence of leukemic transformation has not been determined, as published literature is limited to case reports and small case series. CD5 positivity appears to be even rarer and is only found in a small fraction of DLBCL with leukemic transformation. Treatment regimens for this presentation have not been well-established due to the rarity of the disease and paucity of literature on the subject. Our patient, a 76-year-old female with a history of previously treated stage IIIB follicular lymphoma, was found to have CD5+ DLBCL with leukemic transformation. She was treated with rituximab, cyclophosphamide, doxorubicin, vincristine, and prednisone (R-CHOP) along with intrathecal methotrexate (IT MTX)/cytarabine after CNS involvement was diagnosed. The patient tolerated therapy well, with an objective reduction in leukocytosis and blast count. To our knowledge, this is the first such case of CD5+ DLBCL with leukemic transformation treated with dose-reduced R-CHOP and IT MTX/cytarabine. Her response to therapy indicates that this regimen could be a viable option for the treatment of this exceedingly rare disease presentation.

## Introduction

Diffuse large B-cell lymphoma (DLBCL) is a relatively common subtype of non-Hodgkin’s lymphoma but has a variable presentation. One rare presentation is leukemic transformation, in which malignant cells enter the peripheral circulation, leading to systemic symptoms, a high tumor burden, profound leukocytosis, and a high risk for CNS involvement. When also positive for CD5, this malignancy becomes even more rare and aggressive. We presented here a case with both of these features, occurring in the form of CD5+ DLBCL with leukemic transformation. 

Due to the rarity of this condition, current literature does not have enough evidence as to which treatment regimens lead to optimal patient benefit, though aggressive treatment regimens like rituximab, cyclophosphamide, vincristine, doxorubicin, and dexamethasone (R-hyper-CVAD) have been proposed. Given her age and comorbidities, our patient was deemed too frail for such an aggressive regimen and was treated instead with dose-attenuated rituximab, cyclophosphamide, doxorubicin, vincristine, and prednisone (R-CHOP) and intrathecal methotrexate (IT MTX)/cytarabine once cerebrospinal fluid (CSF) involvement had been confirmed. We found that this regimen did lead to an objective decrease in tumor burden, yielding a significant improvement in both leukocytosis and blast count. To our knowledge, this is the first such case treated in this manner.

## Case presentation

**Figure 1:**
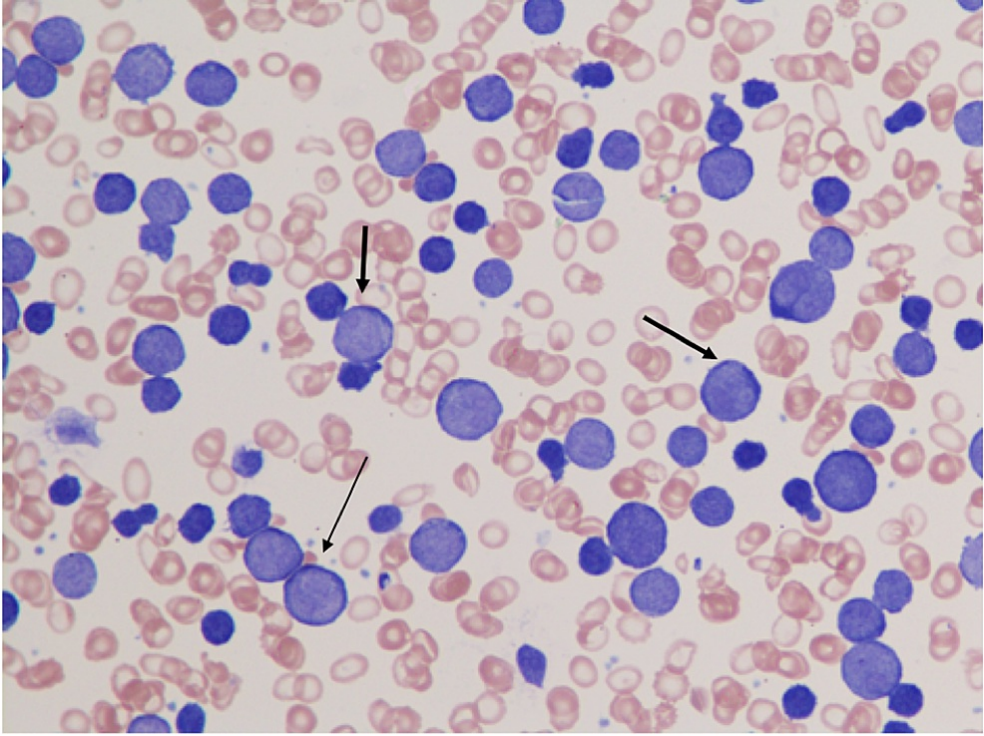
Peripheral blood smear showing leukocytosis and large blast cells.

**Figure 2:**
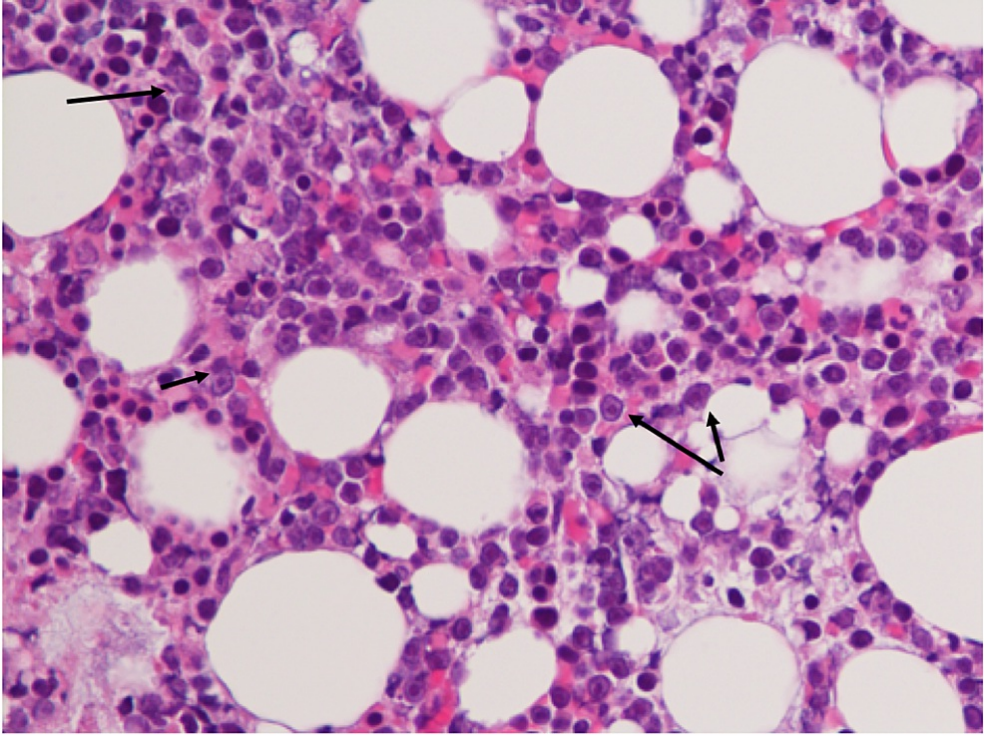
Bone marrow biopsy showing hypercellularity and large aggregates of abnormal cells.

**Figure 3:**
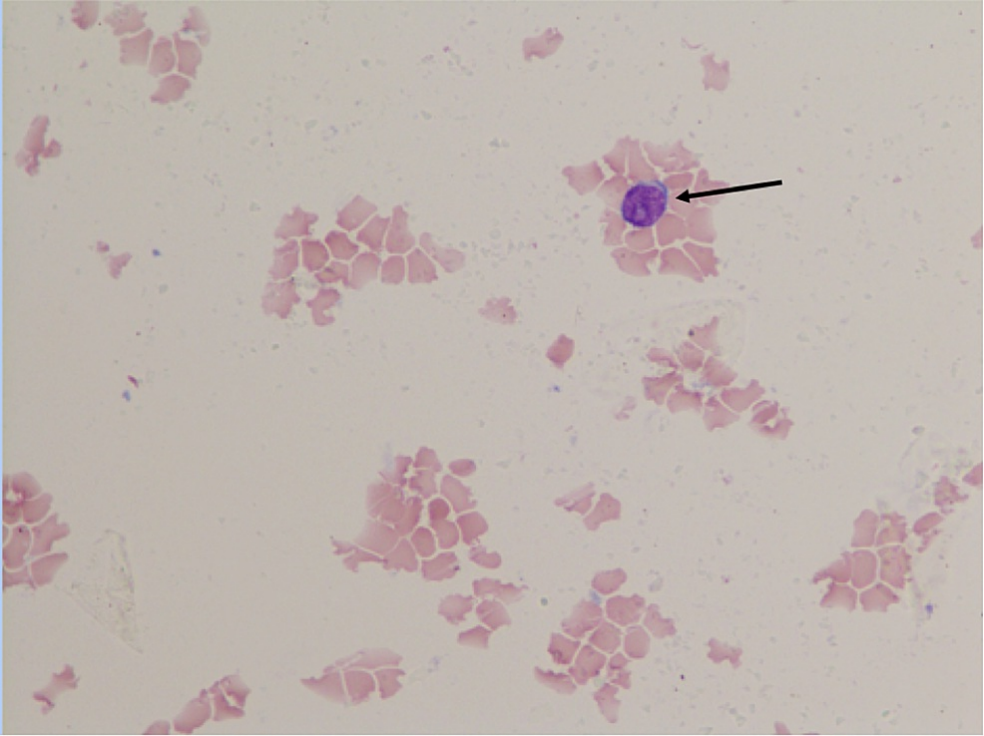
Cerebrospinal fluid showing blast cell infiltration before treatment.

Our patient is a 76-year-old African-American female who presented with fatigue, nausea and vomiting over a period of three days. She had a history of infiltrating ductal carcinoma of the breast, discovered and treated in 2000 with modified radical mastectomy and in remission after five years of tamoxifen therapy. She also had stage IIIB follicular lymphoma, discovered and treated in 2013 with six cycles of bendamustine/rituximab. She completed therapy in January 2014 and, following remission, underwent nine bimonthly cycles of rituximab maintenance therapy. Upon presentation, her leukocyte count was 395,000 with 98% blasts, her hemoglobin (Hgb) was 5.1 g/dL, and her serum potassium was 7.9 mmol/L. Flow cytometry confirmed B-cell malignancy (CD5+, CD10+, CD19+, CD20+, CD34-, TDT-) and large blasts were present on a peripheral smear (Figure 1), along with aggregates of abnormal cells on bone marrow biopsy (Figure 2). Cytogenetic and fluorescence in situ hybridization (FISH) analysis revealed high-grade DLBCL with leukemic transformation (Myc+, IGH+, Bcl-2+). She began systemic chemotherapy with CHOP, dose attenuated by 25% due to patient frailty, and rituximab held initially due to concerns of disease flare and extreme tumor lysis. A lumbar puncture was performed due to a high risk of CNS involvement, which revealed CSF dissemination of disease (Figure 3) and which was later treated with IT MTX alternated with cytarabine. Rituximab was added to the chemotherapy regimen on post-CHOP day six due to persistent leukocytosis. The patient tolerated therapy well, demonstrating an objective decrease in leukocytes (from 395,000 to 15,100) and blast count (from 98% to 17%) prior to discharge, along with a complete absence of blast cells in her CSF. She later completed a second cycle of R-CHOP and IT chemotherapy on an outpatient basis, after which her leukocyte count was 22,850 (87.9% neutrophils, 1.2% granulocytes, 4.0% lymphocytes, 6.6% eosinophils, 0.0% basophils) with no evidence of blasts on peripheral smear or malignant cells on CSF cytometry.

## Discussion

Despite CD5+ DLBCL accounting for 5-10% of all DLBCL cases [1], its progression to the leukemic phase is exceedingly rare. It is unclear why this progression occurs in a small subset of patients. However, one mechanism proposed is that differential expression of adhesion molecules facilitates the migration of lymphocytes from lymph nodes into the systemic circulation [2]. The CD5 positivity is of particular concern, as it has been posited to support B-cell survival and decrease feedback mechanisms that can lead to cell death [3]. It is also more likely to present in an advanced clinical stage after CNS involvement and carries a poorer prognosis due to a less favorable response to chemotherapy [4]. 

Published literature is limited to individual case reports and case series, which prevents a more thorough characterization of incidence and/or prevalence. One study by Yamaguchi et al. revealed four out of 120 (3.3%) CD5+ DLBCL patients presenting with leukemic transformation [5], while another study by Muringampurath-John et al. revealed five out of 29 (17.2%) DLBCL patients with leukemic transformation presenting with CD5 positivity [6]. Both studies highlight the rarity of this disease but were not able to fully quantify it.

The literature is also unclear as to the optimal treatment regimens for the combined presentation of CD5+ DLBCL with leukemic transformation. An increasing number of case reports separately detail both DLBCL with leukemic transformation [7-11] and CD5 positivity [4,12], but mentions of dual presentation have been limited, as have case series and larger studies [5-6]. The authors of these reports, where treatment is discussed, ultimately favor an anthracycline-based treatment regimen, often paired with rituximab, though R-hyper-CVAD with alternating IT MTX/cytarabine [13] has also been reported. 

Two studies were found to be of particular importance to this discussion, as they highlight two established differences in the treatment approach. Muringampurath-John et al., who focused on the leukemic phase of DLBCL, recommended anthracycline-based regimens with rituximab (R-CHOP or similar), finding complete remission (CR) in 54% of patients with a 4-year overall survival (OS) of 50% [6]. However, as mentioned above only five of these treated cases were CD5+ and their outcomes were not stratified given the small sample size. Yamaguchi et al., on the other hand, focused on CD5+ DLBCL and recommended anthracycline-based regimens without rituximab, finding CR in 68% of patients with 5-year OS of 38% [5]. Again, however only two cases in the study were noted to have an immunoblastic phenotype similar to our case and, given the small sample size, those outcomes were not stratified. So although we have readily available data on treatments and outcomes for both leukemic transformation of DLBCL and CD5+ DLBCL, there is a lack of data on the most optimal treatment regimen for a patient presenting with both of these features. We present our case as an illustration of a possible treatment approach with R-CHOP and IT MTX/cytarabine, which for our patient led to rapid debulking and response. Our results, of course, warrant further study and we hope that our report will lead to further discussion regarding optimal treatment for this rare disease presentation.

## Conclusions

CD5+ DLBCL and DLBCL with leukemic transformation are rare presentations of non-Hodgkin’s lymphoma, but both have been reported with increasing regularity and have established treatment regimens. Our report is unique in that the patient presented with an exceedingly rare case of both of these features. CD5+ DLBCL with leukemic transformation is a poorly understood disease without a clear pathophysiologic mechanism and for which a standard of care has not been established. We found that our patient showed a measurable clinical response to dose-attenuated R-CHOP and IT MTX/cytarabine, as evidenced by an absence of blast cells and CSF cytology after two cycles. Further studies are needed, particularly clinical trials, to better care for patients with this diagnosis and to more firmly establish an optimal chemotherapeutic regimen.
